# Luteal Presence and Ovarian Response at the Beginning of a Timed Artificial Insemination Protocol for Lactating Dairy Cows Affect Fertility: A Meta-Analysis

**DOI:** 10.3390/ani10091551

**Published:** 2020-09-02

**Authors:** Stefan Borchardt, Alina Pohl, Wolfgang Heuwieser

**Affiliations:** Clinic for Animal Reproduction, Faculty of Veterinary Medicine, Freie Universität Berlin, Koenigsweg 65, 14163 Berlin, Germany; alina.pohl@fu-berlin.de (A.P.); w.heuwieser@fu-berlin.de (W.H.)

**Keywords:** dairy cow, reproductive management, timed artificial insemination

## Abstract

**Simple Summary:**

The widespread adoption of synchronization protocols for timed artificial insemination (AI) in the dairy industry has improved reproductive performance. The most common timed AI protocol is a combination of two hormones (i.e., gonadotropin-releasing hormone and prostaglandin F 2α). Primarily, these protocols increase the insemination risk without affecting the risk of conception. Based on an advanced understanding of the underlying physiology of timed AI protocols, modifications have been developed in order to increase the conception risk. We performed a meta-analytical assessment to evaluate the effect of luteal presence and ovulatory response at the beginning of a timed AI protocol. There was a clear benefit on conception risk for cows starting a timed AI protocol with a functional corpus luteum (+10.5 percentage units) and cows ovulating at the beginning of a timed AI protocol (+11.0 percentage units). Our work highlights the importance of prerequisites for achieving excellent fertility results in timed AI protocols for lactating dairy cows.

**Abstract:**

Progesterone (P4) concentration during follicular growth has a major impact on fertility response in timed artificial insemination (TAI) protocols. Luteal presence at the beginning of a TAI protocol and ovarian response after the first gonadotropin-releasing hormone (GnRH) injection (G1) affect P4 concentration and subsequently pregnancy per artificial insemination (P/AI). A systematic review of the literature and meta-analytical assessment was performed with the objective of evaluating the magnitude of the effect of luteal presence and ovarian response at the beginning of a TAI protocol on P/AI in lactating dairy cows. We considered only studies using synchronisation protocols consisting of GnRH and prostaglandin F _2α_. The time interval between G1 and prostaglandin F _2α_ (PGF _2α_) had to range from 5 to 7 d. The time interval between the PGF _2α_ injection and G2 had to range from 48 to 72 h. We used 28 controlled experiments from 27 published manuscripts including 16,489 cows with the objective of evaluating the effect size of having a functional corpus luteum (CL) at G1 on P/AI. Information regarding ovulatory response after G1 was available for 5676 cows. In a subset of cows (*n* = 4291), information was available for luteal presence and ovulatory response at the initiation of the TAI protocol. A functional CL at G1 increased (*p* < 0.001) the relative risk of conceiving (RR (relative risk) = 1.32; 95% CI = 1.21–1.45) in lactating dairy cows. Ovulation after G1 increased (*p* < 0.001) the relative risk of conceiving (RR = 1.29; 95% CI = 1.20–1.38) in lactating dairy cows. The effect of ovulatory response on P/AI after G1 was affected by luteal presence at G1. In summary, there was a clear benefit on P/AI for cows starting a TAI protocol with a functional CL (+10.5 percentage units) and cows ovulating at the beginning of a TAI protocol (+11.0 percentage units).

## 1. Introduction

The widespread adoption of synchronisation protocols for timed artificial insemination (TAI) in the dairy industry has improved reproductive performance [1]. The most common TAI protocol is a combination of gonadotropin-releasing hormone (GnRH) and prostaglandin F _2α_ [2]. Primarily, these protocols increase the insemination risk without affecting the risk of conception [3]. An advanced understanding of the underlying physiology of TAI protocols and appropriate modifications has led to an increase in conception risk [4]. The prerequisites for achieving excellent fertility results in TAI protocols for lactating dairy cows are (1) a functional corpus luteum (CL) at the first GnRH injection (G1), (2) ovulation after G1, (3) adequate luteolysis after PGF _2 α_, and (4) timely ovulation after the final GnRH (G2). Providing an optimum progesterone (P4) environment during the preovulatory follicle growth phase is important for fertility [5] and can be obtained by luteal presence at G1 and/or ovulation after G1. This can be achieved when cows were at specific stages of the estrous cycle at the beginning of the TAI protocol [6], which is the rationale for presynchronisation protocols. These protocols can be implemented to increase the proportion of cows in early diestrus at the first GnRH injection of the timed AI protocol for achieving the maximum conception risk [7].

Starting a TAI protocol with a functional CL positively impacts pregnancy per artificial insemination (P/AI) in TAI protocols in multiple studies [8,9]. Cows having an intermediate P4 concentration (P4 ≥ 0.5 ng/mL and <7.0 ng/mL) at G1 had the greatest pregnancy per AI compared with cows having either low (<0.5 ng/mL) or high P4 (≥7.0 ng/mL) concentration using a dataset with 6144 cows [4]. 

It was shown in multiple studies that ovulation to G1 in an Ovsynch protocol improved P/AI [10,11,12]. Ovulation and GnRH-induced LH release are suppressed in the presence of a CL and at larger concentrations of progesterone in dairy cattle [13,14]. Therefore, cows having intermediate P4 concentration at G1 have favorable P/AI [15].

Individual studies often encompass a limited number of herds with similar management practices, climatic conditions, and genetic background that limits external validity. This limits the inference observed for a treatment effect [16]. While it has been shown in multiple individual studies that either the presence of a CL at G1 [8,9] or ovulation after G1 [10,11,12] have a positive effect on P/AI in TAI protocols, there is only a small number of studies evaluating both at the same time. In addition, some studies [17,18,19] indicate that the presence of a CL at the beginning of a TAI protocol had no effect on P/AI.

To address this challenge, this meta-analysis was designed to evaluate the individual and combined effects of luteal presence and ovarian response at the beginning of a TAI protocol on P/AI in lactating dairy cows. The main hypothesis of the present study was that the presence of a corpus luteum at the initiation of a timed AI protocol and ovulation after G1 have a positive effect on fertility.

## 2. Materials and Methods

### 2.1. Literature Search

**Figure 1:**
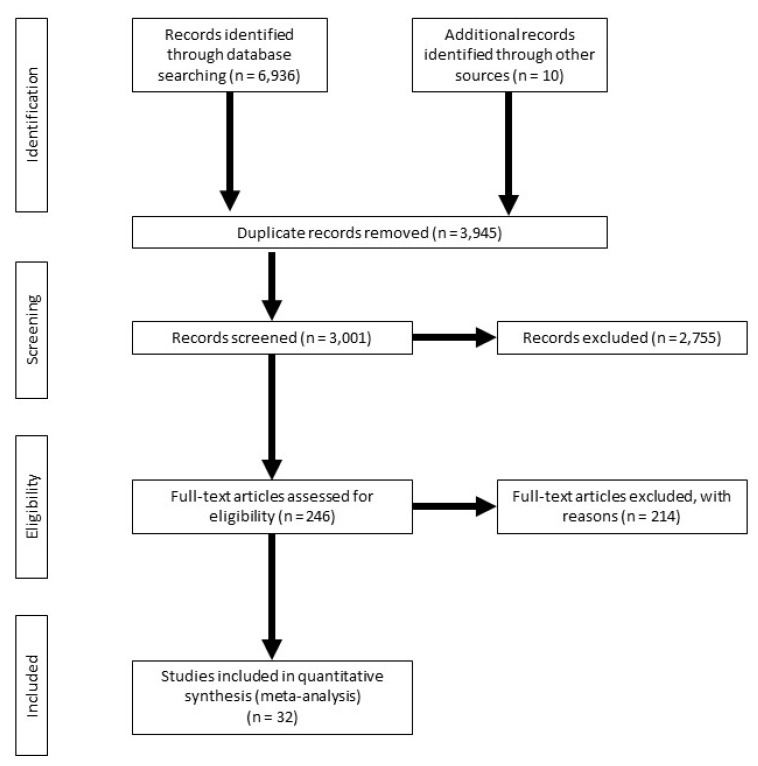
The PRISMA flow diagram [20] of the systematic review from initial search and screening to final selection of publications to be included in the meta-analysis.

The literature search was conducted in PubMed [20], ScienceDirect [21], and Google Scholar [22] using the search terms “dairy cow AND Ovsynch”. Additional manuscripts were obtained directly from researchers in the field of reproductive biology. Results from the online search and personal communications were assessed individually for consideration for the meta-analysis. Figure 1 depicts a PRISMA diagram [23] of the flow of data collection for the meta-analysis. After the initial search and screening, 246 publications were assessed for eligibility. From those, 214 experiments were excluded because of the following reasons: lack of details on timed AI protocols (35 experiments); presence of a CL at or ovulatory response after G1 not evaluated (105 experiments); data on P/AI not reported for individual groups (e.g., CL versus no CL; 35 experiments); exogenous P4 supplementation in all cows (20 experiments); heifers as experimental units (10 experiments); review article or meta-analysis (9 experiments).

### 2.2. Inclusion and Exclusion Criteria

We considered only studies using synchronisation protocols consisting of GnRH and prostaglandin F _2α_. The time interval between G1 and PGF _2α_ had to range from 5 to 7 d. The time interval between the PGF _2α_ injection and G2 had to range from 48 to 72 h. Studies had to report information on either the presence of a functional corpus luteum at G1 and/or ovulatory response after G1. Experimental groups in which exogenous P4 was supplemented between G1 and PGF_2α_ were excluded from the analysis. Experimental groups from studies [12,24,25] in which exogenous estradiol was supplemented were not excluded from the analysis. Based on these criteria, the meta-analysis included a total of 28 manuscripts including 16,489 cows with information on luteal presence at G1, 11 manuscripts including 5361 cows with information on ovulatory response after G1, and 6 manuscripts including 2928 cows with information on both. 

### 2.3. Data Extraction

Data extraction was performed by a single investigator (S. Borchardt) and validated by one coauthor (A. Pohl). For each study, recorded information included authors, year of publication, number of herds, sample size calculation, stratification of results by parity and AI number, GnRH product and dose, PGF _2α_ product and dose, time schedule of the breeding TAI protocol, days in milk at TAI, method to determine the presence of a CL (i.e., blood P4 versus ultrasound), and method to determine ovulation after G1. The relevant information is summarized in Table 1. 

### 2.4. Statistical Analyses

The meta-analysis was conducted using MedCalc (version 15.6.1, MedCalc Software, Mariakerke, Belgium) as described elsewhere [47]. 

As a control group, we either used cows having no functional CL at G1 or cows that did not ovulate after G1. We calculated the relative risk (RR) for being diagnosed pregnant 32 d after TAI using a fixed effects and a random effects model, respectively. Under the fixed effects model, we assumed that all the experimental groups came from a common population and that the effect size regarding P/AI is not significantly different among the different trials as described by the heterogeneity (*I*^2^). However, if there is a significant heterogeneity, the random effects model may be more appropriate, in which both the random variation within the experimental group and the variation between the different experimental groups is incorporated [48]. MedCalc uses the Mantel–Haenszel method for calculating the weighted summary RR under the fixed effects model. The heterogeneity statistic is incorporated to calculate the summary RR under the random effects model [49]. 

Heterogeneity (*I*^2^) is the percentage of observed total variation across studies that is due to real heterogeneity rather than chance. It is calculated as

*I*^2^ = 100% × (Q − df)/Q.

Herein, Q is Cochran’s heterogeneity statistic and df is the degrees of freedom. Negative values of *I*^2^ are put equal to zero so that *I*^2^ lies between 0% and 100%. A value of 0% indicates no observed heterogeneity, and larger values show increasing heterogeneity [50].

Forest plots were used to visually display the estimated effect size, 95% confidence intervals, and study weights. The weight (solid square) and the 95% CI (whiskers) are depicted for each study. The dotted vertical line represents a relative risk of zero or no effect. Squares located on the left side of this line represent studies showing a negative effect of presence of a CL at G1 or ovulation after G1 on P/AI, whereas squares located on the right side of this line indicate a positive effect on P/AI. The overall effect size was summarized into a pooled relative risk using either a fixed or a random effects model.

The presence of publication bias was investigated using funnel plots, which are a simple scatter plot of the treatment effect estimates from individual comparisons plotted against comparison precision. Effect estimates from comparisons with a small number of animal units will scatter more widely at the bottom of the graph, and the spread narrows for those with higher numbers of units. In the absence of bias, the plot should approximately resemble a symmetrical (inverted) funnel. If bias exists, for example because smaller comparisons without statistically significant effects remain unpublished, this will lead to an asymmetrical appearance of the funnel plot, and a gap will be evident in a bottom corner of the graph. In this situation, the effect calculated in a meta-analysis will tend to overestimate the intervention effect. The more pronounced the asymmetry, the more likely it is that the bias will be substantial. 

## 3. Results

### 3.1. Overall Effect of a Functional Corpus Luteum at the First GnRH Injection on P/AI

**Figure 2:**
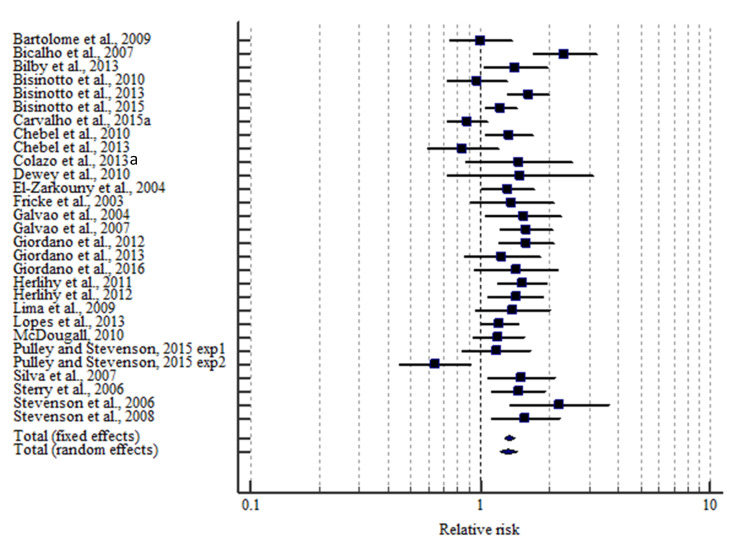
Effect of presence of a functional corpus luteum (CL) at the beginning of a timed AI program on the relative risk of pregnancy on d 32 after AI. The weight (solid square) and the 95% CI (whiskers) are depicted for each study. The overall effect size was summarized into a pooled relative risk using either a fixed or a random effects model. Cows without a functional CL were the reference for comparison.

**Figure 3:**
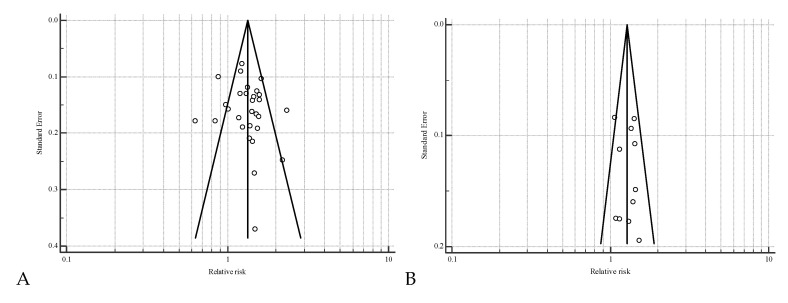
Funnel plots for the risk of pregnancy on d 32 after timed AI in response to the presence of a functional corpus luteum (CL; Panel **A**) or ovulation after the first GnRH (G1) injection at the initiation of the timed AI protocol (Panel **B**). Standard errors are inversely proportional to the number of cows in the study (smaller standard errors represent larger studies). Effect estimates represent the increase in pregnancy per AI associated with the presence of a functional CL or ovulation after G1 (values greater than 0 indicate greater pregnancy per AI in cows with a functional CL or cows that ovulated compared with controls). In the absence of heterogeneity or bias, the plot should approximately resemble a symmetrical (inverted) funnel with studies lying within these lines. For example, if bias is present because smaller treatments without statistically significant effects remain unpublished, this will lead to an asymmetrical appearance of the funnel plot, and a gap will be evident in a bottom corner of the graph.

There were 28 manuscripts available with a controlled study design including 16,489 TAI to evaluate the effect of having a functional CL at G1 on P/AI on d 32 after TAI. A functional CL at G1 increased (*p* < 0.001) the relative risk of conceiving (RR = 1.32; 95% CI = 1.21–1.45) in lactating dairy cows using the random effects model (Figure 2 and Table 2). There was substantial heterogeneity (*I*^2^ = 65.1%; *p* = 0.001) among the 28 experimental groups regarding P/AI. The funnel plot depicted a moderate asymmetry (Figure 3). There were 3 studies showing a slightly negative effect of having a functional CL at G1 [17,18,19]. These studies were only considering resynchronized TAI.

In order to reduce the amount of heterogeneity among the studies, we classified the studies based on service number (first postpartum TAI versus resynchronized TAI), TAI protocol (7d Ovsynch versus other TAI protocols) and definition of a functional CL at G1 (blood P4 versus transrectal ultrasound). Classification of studies based on the service number considerably reduced the amount of heterogeneity among the studies regarding P/AI.

### 3.2. Effect of a Functional Corpus Luteum at the First GnRH Injection on P/AI Depending on the Service Number

Two studies [8,31] did not differentiate between first and second or greater TAI and were not included in this analysis.

For first postpartum service, there were 16 manuscripts available including 8783 TAI to evaluate the effect of having a functional CL at G1 on P/AI. A functional CL at G1 increased (*p* < 0.001) the relative risk of conceiving (RR = 1.48; 95% CI = 1.38–1.60) in lactating dairy cows using the fixed effects model. There was moderate heterogeneity (*I*^2^ = 33.7%; *p* = 0.093) among the 16 studies regarding P/AI. We found no evidence from the funnel plot evaluation of any publication bias for the effect of having a functional CL at G1 for cows receiving their first postpartum TAI.

For resynchronized TAI, there were 12 manuscripts available, including 6103 TAI to evaluate the effect of having a functional CL at G1 on P/AI on 32 after TAI. A functional CL at G1 increased (*p* < 0.064) the relative risk of conceiving (RR = 1.18; 95% CI = 0.99–1.40) in lactating dairy cows using the random effects model. There was substantial heterogeneity (*I*^2^ = 73.1%; *p* = 0.001) among the 12 studies regarding P/AI. The funnel plot depicted a moderate asymmetry for the effect size in cows receiving their resynchronized TAI.

### 3.3. Overall Effect of Ovulation after the First GnRH Injection on P/AI

**Figure 4:**
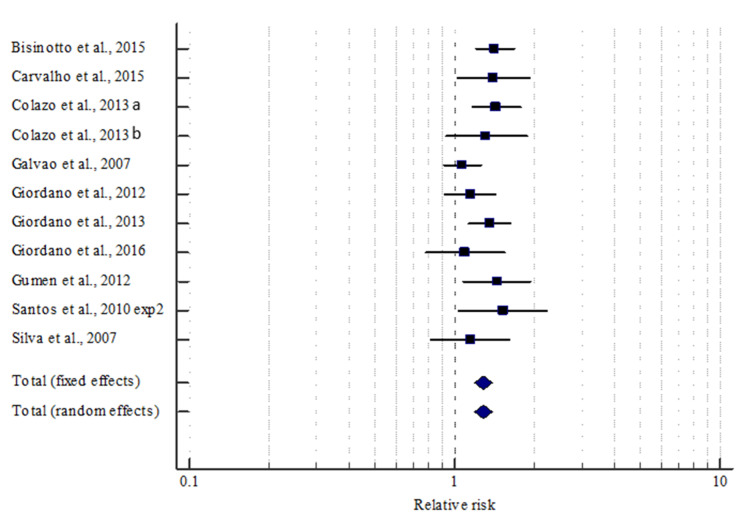
Of ovulation after the first GnRH injection (G1) in a timed AI program on the odds of pregnancy on d 32 after AI. The weight (solid square) and the 95% CI (whiskers) are depicted for each study. The overall effect size was summarized into a pooled relative risk using either a fixed or a random effects model. Cows without ovulation after G1 were the reference for comparison.

There were 11 manuscripts available with a controlled study design including 5,676 TAI to evaluate the effect of ovulation after G1 on P/AI on d 32 after TAI. Ovulation after G1 increased (*p* < 0.001) the relative risk of conceiving (RR = 1.29; 95% CI = 1.20–1.38) in lactating dairy cows using the fixed effects model (Figure 4 and Table 3). There was no heterogeneity (*I*^2^ = 12.6 %; *p* = 0.324) among the 11 studies regarding P/AI. We found no evidence from funnel plot evaluation of any publication bias for the effect of ovulation after G1 on P/AI (Figure 3).

### 3.4. Effect of Ovulation after the First GnRH Injection on P/AI Depending on the Presence of a CL at G1

Five studies [10,32,37,42,43] did not differentiate between luteal presence at G1 and ovulation after G1 concurrently. These studies were excluded from the analysis.

For cows with a CL at G1, there were 6 manuscripts [8,9,11,12,31,36] available, including 3121 timed AI to evaluate the effect of ovulation after G1 on P/AI on d 32 after TAI. Ovulation after G1 in these cows increased (*p* < 0.001) the relative risk of conceiving (RR = 1.20; 95% CI = 1.10–1.31) on d 32 after TAI using the fixed effects model. There was no heterogeneity (*I*^2^ = 0.0%; *p* = 0.675) among the 6 studies regarding P/AI.

For cows without a CL at G1, there were 6 manuscripts [8,9,11,12,31,36] available including 1048 timed AI to evaluate the effect of ovulation after G1 on P/AI on d 32 after TAI. Ovulation after G1 in these cows increased (*p* < 0.001) the relative risk of conceiving (RR = 2.50; 95% CI = 1.92–3.24) on d 32 after TAI using the fixed effects model. There was no heterogeneity (*I*^2^ = 0.0%; *p* = 0.496) among the 6 studies regarding P/AI.

**Figure 5:**
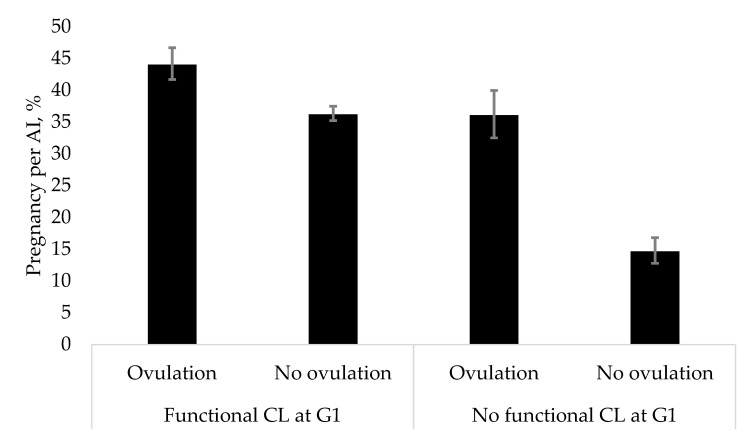
Overall proportion (95% CI) of pregnancy per artificial insemination (P/AI) in 6 experimental groups depending on luteal presence at the first GnRH injection (G1) and ovarian response after G1. There were 3121 timed AI (left side) from cows having a functional corpus luteum (CL) at G1 (ovulation after G1 *n* = 1398; no ovulation after G1 *n* = 1723) and 1170 timed AI (right side) from cows having no functional corpus luteum at G1 (Ovulation after G1 *n* = 806; no ovulation after G1 *n* = 364). The effect size for P/AI was estimated using the fixed or random effects model depending on the heterogeneity among the studies. For cows with a CL at G1, ovulation after G1 increased (*p* < 0.001) the relative risk of conceiving (RR = 1.20; 95% CI = 1.10–1.31) on d 32 after TAI using the fixed effects model. For cows without a CL at G1, ovulation after increased (*p* < 0.001) the relative risk of conceiving (RR = 2.50; 95% CI = 1.92–3.24) on d 32 after TAI using the fixed effects model.

The interaction of presence of a CL at G1 and ovarian response after G1 was further analyzed using the fixed and random effects models to estimate the proportion of P/AI for each of the 4 groups (Figure 5). Cows with a functional CL at G1 that ovulated after G1 (*n* = 1398) had the highest overall P/AI (44.2%; 95% CI = 39.2–49.2%). Cows with a functional CL at G1 that did not ovulate (*n* = 1723; 36.4%; 95% CI = 34.1–38.7%) and cows without a functional CL at G1 that ovulated (*n* = 806; 36.2%; 95% CI = 29.1–43.7%) had intermediate overall P/AI. Cows without a functional CL at G1 that did not ovulate (*n* = 364) had the lowest overall P/AI (14.8%; 95 % CI = 11.4–18.8%).

## 4. Discussion

The results from the present study indicate that luteal presence and ovarian response at the beginning of a TAI protocol have a positive effect on P/AI. The effect of luteal presence at G1 on P/AI was more pronounced in cows receiving their first TAI. There was also a synergistic effect of luteal presence at G1 and ovulation after G1 on P/AI.

Understanding the limitations of TAI protocols consisting of GnRH and PGF _2α_ is critical to maximize its efficiency [51]. Requirements for maximum P/AI in such TAI protocols includes (1) growth of the follicle during an adequate P4 environment either by inducing the ovulation of a dominant follicle after the first GnRH treatment [10,11,12] and/or by having a functional CL present at G1 [51], (2) inducing complete luteolysis in response to PGF _2α_ treatment as evidenced by P4 concentrations < 0.4 ng/mL [4], and (3) timely ovulation of the preovulatory follicle in response to G2 [51,52].

Ovulation of a dominant follicle after G1 improved P/AI (RR = 1.29) by approximately 11 percentage units in this meta-analysis. Ovulatory response to G1 is dependent on the stage of the estrous cycle at which GnRH is administered with cows on d 5 to 9 of the estrous cycle having the greatest ovulatory response [6]. Therefore, presynchronisation protocols (i.e., Presynch–Ovsynch, G6G, Double–Ovsynch) have been implemented to increase the proportion of cows in early diestrus at G1 and thereby increase the risk of ovulation and ultimately P/AI [7]. Interestingly, the ovulatory response to G1 was decreased when P4 concentrations were above 3 ng/mL because of a negative effect of P4 on the GnRH-induced LH surge [13,15]. Progesterone may directly inhibit LH release at the pituitary gland because receptors for progesterone are present in the bovine pituitary [53]. Furthermore, GnRH can regulate its own receptor with greater GnRH release by upregulating the GnRH receptor in the anterior pituitary [54]. Therefore, the progesterone inhibition of GnRH pulses may decrease the number of pituitary GnRH receptors and responsiveness of the pituitary to GnRH. This is in agreement with our study. Cows with a functional CL at G1 had a lower ovulatory risk (44.8%; 1398/3121) compared with cows lacking a functional CL at G1 (68.8%; 806/1170).

Having a functional CL at G1 improved P/AI (RR = 1.32) by approximately 10 percentage units in this meta-analysis. Exposure to luteal concentrations of P4 during growth of the dominant follicle influences subsequent fertility. Lactating dairy cows in mid-diestrus usually have P4 concentrations that range from 3 to 6 ng/mL [8,29,55]. The ideal concentration of P4 during follicle growth that maximizes fertility remains unknown [56]. Evidence from an experiment in which P4 concentration was manipulated suggests that a minimum of 2 to 3 ng/mL are needed to achieve similar P/AI in cows lacking a functional CL compared with cows having a functional CL [8]. The exact mechanism needs to be determined, but it is suggested that low concentrations of P4 influence follicle and oocyte development through an increased secretion of LH that accelerates follicle growth and can depress oocyte quality [56]. Furthermore, cows lacking a functional CL at G1 and ovulating a dominant follicle after G1 are more prone to incomplete luteolysis. However, the lack of luteolytic response from the new CL seems to be conditional on the co-existence of an older CL already present at G1 that is mature enough to undergo luteolysis after treatment with exogenous PGF _2α_. One strategy to improve complete luteolysis is to administer 2 injections of PGF _2α_ 24 h apart [47]. The benefit of a second PGF _2α_ treatment might be restricted to cows with only a G1-induced CL at the time of PGF injection. In a recent study [51], incomplete luteolysis in response to one PGF _2α_ treatment was less prevalent when a more mature CL was co-existent with the new G1 induced CL (old CL + new CL 9.9% versus only new CL 15.7%). 

In our study, the effect size of having a functional CL on P/AI was conditional on service number. Cows receiving their first TAI showed a greater benefit (RR = 1.48; P/AI CL+ 42.9% versus CL 29.2%) from having a functional CL compared with cows receiving a subsequent TAI (RR = 1.18; P/AI CL+ 37.1% versus CL 30.6%). For the first TAI, most of the studies were using a presynchronisation strategy consisting either of two PGF _2α_ treatments administered 14 d apart with the second treatment administered 9 to 14 d before initiation of the Ovsynch protocol (i.e., Presynch–Ovsynch) or a combination of GnRH and PGF _2α_ 6 to 7 d before initiation of the Ovsynch protocol (i.e., G6G, Double–Ovsynch). The greater effect size of a functional CL at G1 for cows receiving their first TAI is underlining the importance of presynchronisation protocols for subsequent fertility. These protocols have been implemented to increase the proportion of cows in early diestrus at G1 to increase the risk of ovulation and the percentage of cows with a functional CL. Lacking a functional CL at G1 is an indicator of cows with poor fertility. Treatment of these cows with exogenous P4 for 7 d in an Ovsynch protocol increased fertility at first TAI as well as for resynchronized TAI [8,28,30]. In a recent meta-analysis [57], the benefit of progesterone supplementation using a single intravaginal insert was observed mainly in cows lacking a CL at the initiation of the timed AI protocol (RR = 1.18; 95% CI = 1.07–1.30) rather than those with a CL (RR = 1.06; 95% CI = 0.99–1.12). Progesterone supplementation with 2 intravaginal inserts restored fertility in lactating dairy cows lacking a CL at the initiation of the timed AI protocol similar to that of cows in diestrus using either a 5 d Cosynch protocol [29] or a 7 d Ovsynch protocol [8].

Although multiple studies showed that ovulation after G1 in an Ovsynch protocol improved P/AI [10,11,12], there is some debate whether this positive effect is limited to cows lacking a functional CL at G1 [9]. This assumption was supported by recent evidence indicating that the concentration of P4 during follicular development was more important for improving P/AI than ovulation after G1 [51]. In our study, the effect size of ovulation after G1 on P/AI was conditional on the presence of a functional CL at G1. Cows lacking a functional CL showed a greater benefit (RR = 2.50; P/AI in ovulatory cows 36.2% versus non-ovulatory cows 14.8%) from ovulation compared with cows bearing a functional CL (RR = 1.20; P/AI in ovulatory cows 44.2% versus non-ovulatory cows 36.4%). Nevertheless, there was still a benefit from ovulation in cows bearing a functional CL at G1. This clearly shows that there is a synergistic effect of ovulation after G1 and luteal presence at G1. Besides an effect of ovulation on circulating P4 concentrations during ovulatory growth, there might be also an effect on proper timing of a new follicle wave resulting in the ovulatory follicle. The emergence of a new follicular wave occurs within 1 d after ovulation, leading to a better control of the age and size of the ovulatory follicle at G2 [58]. Besides presynchronisation protocols, attempts to improve the ovulatory response after G1 in a Double–Ovsynch protocol by either increasing the GnRH dose [11] or administering half the PGF _2 α_ dose (12.5 mg) 2 d before G1 [10] showed a positive effect on ovulation rate (Carvalho et al. [10]: + 20.8%; Giordano et al. [11]: +9.1%) but failed to improve P/AI. However, the possibility of a Type II error (declaring no difference between groups when a difference does exist) must be considered when interpreting data from these studies. The likelihood of detecting a treatment effect on fertility was limited because of only a modest improvement of P/AI for cows ovulating after G1 compared with non-ovulatory cows (Carvalho et al. [10]: +16.4%; Giordano et al. [11]: + 13.7%).

It has been shown in multiple individual studies that presence of a CL at G1 [8,27,28,37] had a positive effect on P/AI in TAI protocols, which is the basis for presynchronisation protocols. However, after a systematic review, there were 3 out of 28 studies [17,18,19] indicating that the presence of a CL at the beginning of TAI protocol had no effect on P/AI. Using a meta-analytical assessment, we were able to better describe the effect size of a CL at G1 on P/AI and some additional factors such as ovulation after G1 and insemination number, which modulated this effect size. 

## 5. Conclusions

Based on a meta-analytical assessment including 17,610 cows, we were able to show that luteal presence and ovarian response at the beginning of a TAI protocol enhance fertility in lactating dairy cows subjected to TAI protocols. Ovulation of a dominant follicle after G1 and the presence of a functional CL at G1 improved P/AI by approximately 11 and 10 percentage units, respectively. The fertility response might be achieved by optimizing the hormonal environment during follicular growth and improving the synchronisation rate. The benefit of ovulatory response was observed largely in cows lacking a functional CL at G1. Nevertheless, ovulation after G1 was also beneficial in cows bearing a functional CL at G1. The effect size of having a functional CL at the initiation of a TAI protocol was more pronounced in cows receiving their first postpartum TAI compared with resynchronized TAI. These results underline the importance of ovarian physiology to maximize fertility in TAI protocols.

## Figures and Tables

**Table 1:** Summary of manuscripts (*n* = 32) evaluating the effect of luteal presence at the first gonadotropin-releasing hormone (GnRH) injection (G1) and/or ovulation after G1 in timed artificial insemination (TAI) protocols.

Study	No. of Cows	No. of Herds	CL at G1 ^1^	Ovu. at G1 ^2^	AI no. ^3^	Presynch ^4^	TAI Protocol ^5^
Bartolome et al. [26]	333	1	P4 1 ng/mL		1	PO	7d Cosynch 72
Bicalho et al. [27]	801	5	P4 1 ng/mL		1	PO	7d Ovsynch
Bilby et al. [28]	579	5	US		2+		7d Ovsynch
Bisinotto et al. [5]	283	1	US		2+		5d Cosynch 72
Bisinotto et al. [29]	1180	1	US 10 mm		1/2+	PO	5d Cosynch 72
Bisinotto et al. [8]	1289	5	US 20 mm	US	1/2+	PO	7d Ovsynch
Carvalho et al. [18]	818	1	P4 1 ng/mL		2+		7d Ovsynch
Carvalho et al. [10]	220	2		US	1	DO	7d Ovsynch
Chebel et al. [30]	787	7	P4 1 ng/mL		1		7d Ovsynch
Chebel et al. [17]	224	2	US		2+	GnRH/PGF _2 α_	7d Ovsynch/Cosynch 48
Colazo et al. [31]	608	3	P4	US	1/2+		7d Ovsynch
Colazo et al. [32]	241	1		US	1	PO	7d Ovsynch
Dewey et al. [33]	221	2	P4 1 ng/mL		2+		7d Cosynch 72
El-Zarkouny et al. [34]	402	2	P4 1 ng/mL		1	PO	7d Ovsynch
Fricke et al. [35]	264	1	US 10 mm		2+		7d Cosynch 48
Galvao et al. [24]	312	2	P4 1 ng/mL		1	PO	8d ECP Synch
Galvao et al. [12]	1000	1	US	US	1	PO	7d ECP Synch
Giordano et al. [9]	741	1	P4 1 ng/mL	US	2+	DO	7d Ovsynch
Giordano et al. [11]	651	1	P4 1 ng/mL	US	1	DO	7d Ovsynch
Giordano et al. [36]	268	4	P4 1 ng/mL	US	1	PO	7d Ovsynch
Gumen et al. [37]	329	1		US	1	PO	7d Ovsynch
Herlihy et al. [38]	407	8	P4 1 ng/mL		1		7d Ovsynch
Herlihy et al. [39]	739	3	P4 0.5 ng/mL		1	PO/DO	7d Ovsynch
Lima et al. [25]	431	2	P4 1 ng/mL		1	PO	8d ECP Synch
Lopes et al. [40]	1107	1	P4 1 ng/mL		2+	GnRH	7d Ovsynch
McDougall [41]	546	12	P4 1 ng/mL		1		7d Ovsynch
Pulley and Stevenson [19]	669	1	P4 1 ng/mL		2+	GnRH	5d Ovsynch
Santos et al. [42]	333	2		US	1	PO	5d Cosynch 72/ 7d Cosynch 72
Silva et al. [43]	504	1	P4 1 ng/mL	US	2+	PGF_2α_	7d Ovsynch
Sterry et al. [44]	381	2	US 10 mm		1	PO	7d Cosynch
Stevenson et al. [45]	321	6	P4 1 ng/mL		1/2+		7d Ovsynch
Stevenson et al. [46]	915	6	US		1	PO	7d Ovsynch

^1^ CL at G1 = presence of a corpus luteum at the beginning of the TAI protocol was determined either by transrectal ultrasound (US; threshold in mm diameter if appropriate) or by evaluating blood progesterone (P4; threshold in ng P4/mL if appropriate) concentration.^2^ Ovu. at G1 = ovulatory response after G1 was determined either by transrectal ultrasound (US) or by evaluating blood progesterone (P4) concentration. ^3^ AI no. = timed AI was either the first postpartum TAI (1) or a subsequent resynchronized TAI (2+). ^4^ Presynch = hormonal treatments prior to the breeding portion of the TAI protocol are considered as presynchronisation. ^5^ TAI protocol = timed AI protocol to breed cows.

**Table 2:** Effect of having a functional corpus luteum (CL) at the first GnRH injection (G1) of a timed AI protocol using GnRH and prostaglandin F_2α_ on pregnancy per AI d 32 after TAI considering 28 manuscripts.

Manuscript	CL+ ^1^	CL− ^2^	RR ^3^	95% CI	*p*
Bartolome et al. [26]	52/158	57/175	1.01	0.742–1.37	
Bicalho et al. [27]	259/609	35/192	2.33	1.71–3.19	
Bilby et al. [28]	138/422	36/157	1.43	1.04–1.96	
Bisinotto et al. [5]	88/204	35/79	0.97	0.73–1.30	
Bisinotto et al. [29]	472/946	72/234	1.62	1.32–1.98	
Bisinotto et al. [8]	246/640	203/649	1.23	1.06–1.43	
Carvalho et al. [18]	209/601	86/217	0.87	0.72–1.07	
Chebel et al. [30]	192/534	68/253	1.33	1.06–1.69	
Chebel et al. [17]	63/168	25/56	0.84	0.59–1.19	
Colazo et al. [31]	83/259	12/55	1.47	0.86–2.50	
Dewey et al. [33]	43/178	7/43	1.48	0.72–3.07	
El-Zarkouny et al. [34]	123/260	51/142	1.32	1.02–1.70	
Fricke et al. [35]	76/194	20/70	1.37	0.91–2.07	
Galvao et al. [24]	101/231	23/81	1.54	1.06–2.24	
Galvao et al. [12]	323/809	48/191	1.59	1.23–2.06	
Giordano et al. [9]	176/514	49/227	1.59	1.20–2.09	
Giordano et al. [11]	288/604	18/47	1.24	0.86–1.81	
Giordano et al. [36]	70/190	20/78	1.44	0.94–2.20	
Herlihy et al. [38]	149/269	50/138	1.53	1.19–1.95	
Herlihy et al. [39]	287/624	37/115	1.43	1.08–1.89	
Lima et al. [25]	131/344	24/87	1.38	0.96–1.99	
Lopes et al. [40]	293/758	111/349	1.21	1.02–1.45	
McDougall [41]	56/151	122/395	1.20	0.93–1.55	
Pulley and Stevenson [19] exp1	121/354	29/100	1.18	0.84–1.65	
Pulley and Stevenson [19] exp2	47/153	30/62	0.63	0.45–0.90	
Silva et al. [43]	142/376	32/128	1.51	1.09–2.09	
Sterry et al. [44]	153/277	40/106	1.46	1.12–1.91	
Stevenson et al. [45]	92/236	15/85	2.21	1.36–3.59	
Stevenson et al. [46]	303/799	28/116	1.57	1.12–2.19	
*I*^2^ = 65.1 % (*p* = 0.001)					
Total (fixed effects)	4776/11,862	1383/4627	1.34	1.27–1.41	0.001
Total (random effects)	4776/11,862	1383/4627	1.32	1.21–1.45	0.001

*I*^2^ = Proportion of total variation of effect size estimates that is due to heterogeneity. ^1^ Functional CL at G1 determined either by rectal ultrasound or blood progesterone concentration. ^2^ No functional CL at G1 determined either by rectal ultrasound or blood progesterone concentration. ^3^ Relative risk for conceiving at timed AI for cows having either a functional CL (CL+) or no functional CL (CL−) at G1.

**Table 3:** Effect of ovulation after the first GnRH injection (G1) of a timed AI protocol using GnRH and prostaglandin F _2α_ on pregnancy per AI d 32 after TAI considering 11 manuscripts.

Manuscript	Ovulation	No Ovulation	RR ^1^	95% CI	*p*
Bisinotto et al. [8]	268/600	133/423	1.42	1.20–1.68	
Carvalho et al. [10]	89/153	28/67	1.39	1.02–1.90	
Colazo et al. [31]	93/209	124/399	1.43	1.16–1.77	
Colazo et al. [32]	64/149	30/92	1.32	0.93–1.86	
Galvao et al. [12]	214/560	157/440	1.07	0.91–1.26	
Giordano et al. [9]	93/282	132/459	1.15	0.92–1.43	
Giordano et al. [11]	211/404	95/247	1.36	1.13–1.63	
Giordano et al. [36]	52/149	38/119	1.09	0.78–1.54	
Gumen et al. [37]	139/247	32/82	1.44	1.08–1.93	
Santos et al. [42] exp2	78/221	26/112	1.52	1.04–2.23	
Silva et al. [43]	49/137	39/125	1.15	0.81–1.62	
*I*^2^ = 12.6 % (*p* = 0.324)					
Total (fixed effects)	1350/3111	834/2565	1.29	1.20–1.38	0.001
Total (random effects)	1350/3111	834/2565	1.29	1.19–1.39	0.001

*I*^2^ = Proportion of total variation of effect size estimates that is due to heterogeneity. ^1^ Relative risk for conceiving at timed AI for cows either ovulating or not ovulating after the first GnRH injection.
